# From is to ought, and back: how normative concerns foster progress in reasoning research

**DOI:** 10.3389/fpsyg.2014.00219

**Published:** 2014-03-13

**Authors:** Vincenzo Crupi, Vittorio Girotto

**Affiliations:** ^1^Department of Philosophy and Education, University of TurinTurin, Italy; ^2^Center for Experimental Research in Management and Economics, University IUAV of VeniceVenice, Italy

**Keywords:** rationality, reasoning, selection task, pseudodiagnosticity, conjunction fallacy

## Introduction

Can the issue of human (ir)rationality contribute to the scientific study of reasoning? A tempting line of argument seems to indicate that it can't. Here it is. (i) To discuss diagnoses of (ir)rationality arising from research in the psychology of reasoning one has to deal with *arbitration*, i.e., the assessment of competing theories of what a reasoner ought to do, if rational. But (ii), by the Humean divide between *is* and *ought*, arbitration is logically independent from the description of reasoning. And clearly (iii) the main goal of psychological inquiry is just such a description. It follows that normative concerns about diagnoses of (ir)rationality cannot serve the proper scientific purposes of the psychology of reasoning, and would better be left aside altogether in this area. A recent cornerstone for this debate is Elqayam and Evans (2011). Part of their discussion is devoted to voice precisely this criticism of “normativism,” thus favoring a purely “descriptivist” approach in the study of human thinking. In our view, the above argument is essentially valid, but unsound. Premise (i), in particular, may have seemed obvious but doesn't hold on closer inspection, as we mean to show.

In reasoning experiments, participants are assumed to rely on some amount of information, or data, *D*. These include elements explicitly provided (e.g., a cover story), but possibly also further background assumptions. Note that, as a rule, *D* is *not* already framed in a technical language such as that of, say, probability theory: cover stories and experimental scenarios are predominantly verbal in nature, although they may embed more formal fragments (e.g., some statistical information). On the basis of *D*, participants then have to produce one among a set of possible responses *R*, for instance an item chosen in a set of options or an estimate in a range of values allowed (say, 0 to 100%). Here again, the possible responses do *not* belong to a particular formal jargon (although, again, some formal bits may occur in the elements of *R*).

Suppose that some particular response *r* in *R* turns out to be widespread among human reasoners and is said to be irrational. Such a diagnosis, we submit, has to rely on *four* premises. (i) First, one has to identify a formal theory of reasoning *T* as having normative force^1^. (ii) Second, one has to map *D* onto a formalized counterpart D^*^ belonging to the technical language employed in *T*. (iii) Third, one has to map *R*, too, onto a formalized counterpart R^*^ belonging to the technical language of *T*. This step implies, in particular, that the target response *r* within *R* be translated into its appropriate counterpart r^*^. (iv) And finally, one has to show that, given D^*^, r^*^ does contradict *T*. If either of (i)–(iv) is rejected, the charge of irrationality fails. We thus have a classification of the ways in which one can question diagnoses of irrationality that may be attached to the results of a reasoning experiment. Depending on whether (i), (ii), (iii), or (iv) is the main focus of controversy, we will talk about *arbitration*, *data mismatch*, *response mismatch*, and *norm misapplication*, respectively. Relying on this partition, let us now consider three prominent cases in which normative concerns have entered psychological research on reasoning.

## Exhibit 1: the selection task and data mismatch

The debate on Wason's selection task is said to have sparked the rise of a new paradigm in the psychology of reasoning (see, e.g., Over, 2009), and so it seems a primary example of how progress in this field can intertwine with diverging diagnoses of rational behavior (see Sperber et al., 1995, though, for cautionary considerations). In the standard version of the selection task, four cards are employed which have a letter on one side and a number on the other side. One can see the letter-side of two cards (A and C, say), and the number-side of the other two (4 and 7, say). Which of these cards would one need to turn over to decide whether the following statement is true or false? “If there is a vowel on the one side, then there is an even number on the other side.” In the classical analysis of the selection task, this statement was interpreted as a material conditional and referred to the four cards only. The statement would then be true unless some of the four cards has a vowel and an odd number. Accordingly, the A and the 7 cards ought to be turned over; the C and the 4 cards are of no use, logically. Participants often selected the 4 card, largely disregarding the 7 card, and were thus charged of being irrational.

In Oaksford and Chater's (1994, 2003) work, however, the ordinary language sentence “if vowel, then even number” is not taken as a material conditional, but rather as such that its probability is the conditional probability that the card has an even number on one side given that it has a vowel on the other side. Moreover, the conditional statement is referred to a larger deck of which the four cards only represent a sample and in which, finally, the occurrence of both vowels and even numbers are assumed to be relatively rare. This radically different formal reconstruction of the data *D* defining the problem has important consequences. The implication that, for instance, turning over a card showing number 4 is irrational does not hold anymore and an alternative normative analysis is required (see Fitelson and Hawthorne, 2010). In our current terms, the key point of this debate is a matter of *data mismatch*. Importantly, no doubt needs to be raised against the normative status of classical logic to make sense of this case. (A parallel account could be given for non-probabilistic approaches such as Stenning and van Lambalgen's 2008).

## Exhibit 2: the conjunction fallacy and response mismatch

Upon experimental investigation, individuals often rank a conjunctive statement “*x* and *y*” as more probable than one of the conjuncts (e.g., *x*). For instance, most physicians judge that a patient who had pulmonary embolism is more likely to experience “dyspnea and hemiparesis” than “hemiparesis.” Tversky and Kahneman (1983) famously labeled this a fallacy, because in probability theory *Pr*(*x* ∧ *y*) = *Pr*(*x*) for any *x*, *y*, regardless of what information may be available. Note that the latter clause prevents rescue of the rationality of human judgment by an appeal to data mismatch. In fact, in debates about the conjunction fallacy, it is *response mismatch* that has been relentlessly discussed. Given how fundamental and startling this judgment bias seemed, almost all conceivable worries have been aired over the years. Maybe, in the presence of a conjunctive statement “*x* and *y*,” pragmatic considerations led participants to treat the isolated conjunct “*x*” as “*x* ∧ not-*y*.” Or maybe the ordinary language conjunction “*x* and *y*” was mapped onto a logical disjunction (“*x* ∨ *y*”), or a conditional expression (“*y*, assuming that *x*”). Or the quantities to be ranked were not meant to be *Pr*(*x* ∧ *y*) and *Pr*(*x*) because the reference of the ordinary language term “probable” eluded the basic properties of mathematical probability. In each of these cases, the suggested rendition r^*^ of the modal response *r* (here: that statement “*x* and *y*” was more probable than “*x*”) would have *not* contradicted probability theory, thus deflating the charge of irrationality.

Here again, there is no logical reason to saddle this debate with any subtlety concerning the normative appeal of the target formal theory (classical probability) for human reasoning. And while all of the above worries of response mismatch had been already addressed by Tversky and Kahneman (1983) (see, e.g., Girotto, 2011), their recurrent appearance in the literature spurred the development of more and more refined experimental techniques leading to a better understanding of this reasoning bias. (See Wedell and Moro, 2008; Tentori and Crupi, 2012, 2013; Tentori et al., 2013 for discussions).

## Exhibit 3: pseudodiagnosticity and norm misapplication

In its simplest form (e.g., Kern and Doherty, 1982), so-called pseudodiagnosticity task provides participants with a binary set of blank and equiprobable hypotheses *h* and ¬*h* (e.g., two abstract diagnoses), two pieces of evidence *e* and *f* (e.g., two symptoms) and one likelihood value, such as *Pr*(*e*|*h*) = 65%. Participants have to select the most useful among three further likelihood values, *Pr*(*e*|¬*h*), *Pr*(*f*|*h*), and *Pr*(*f*|¬*h*). In the classical interpretation of this phenomenon, participants were said to have “actively chose[*n*] irrelevant information [namely, *Pr*(*f*|*h*)] and ignored relevant information [namely, *Pr*(*e*|¬*h*)] which was equally easily available” (Doherty et al., 1979, p. 119). The standard Bayesian framework was taken as a benchmark theory sanctioning this conclusion. But the idea of so-called pseudodiagnosticity bias was seen by Crupi et al. (2009) as a case of *norm misapplication*.

Crupi et al. (2009) offered formal renditions (D^*^ and R^*^, in our notation) of the experimental scenario (*D*) and the response set (*R*) that were consistent with the classical reading of the task (so they argued on the basis of textual evidence). Thus no data or response mismatch was invoked, in our current terms. Crupi et al., submitted, instead, that the relevant norms of reasoning had been misapplied in the standard interpretation: far from contradicting the benchmark theory, the appropriate formal counterpart r^*^ of the participants' modal response *r* in pseudodiagnosticity experiments turns out to be actually optimal for a Bayesian agent (given D^*^). Tweeney et al. (2010), in turn, criticized this conclusion. However, they outlined themselves a further novel theoretical analysis of the task and did *not* try to revive the once popular interpretation of the phenomenon in its original form. To the extent that the latter is now judged inadequate by all parties involved, at least some theoretical progress was made whatever the outcome of this debate.

## Concluding remarks

According to a seductive argument, debates on the (ir)rationality of participants' responses are better left out of the psychologist's outlook for they would invariably led her to plod on the shaky ground of arbitration. We have challenged this assumption by means of three key examples. The selection task, the conjunction fallacy and pseudodiagnosticity have been extensively investigated in the psychology of reasoning, and all raised lively controversies about human rationality. Yet, issues of arbitration hardly played any substantive role. Once properly reconstructed, the relevant problem was not whether it is rational to depart from the implications of allegedly compelling normative theories such as logic or probability theory. Instead, much of the research done with these classical paradigms was focussed on whether and how those implications could connect with observed behavior given that data mismatch, response mismatch or norm misapplication may have occurred.

Arbitration between competing norms of reasoning is central to certain areas of philosophy but remains marginal in psychological research, and for good reasons, loosely related to the so-called *is*/*ought* divide: arbitration does require specific forms of argumentation that lie outside the usual scope of empirical research (see, e.g., Schurz, 2011; Pettigrew, 2013). Concerns of data mismatch, response mismatch and norm misapplication, on the contrary, are amenable to independent scrutiny in purely descriptive terms (be that at the empirical or theoretical level). Sometimes earlier charges of irrationality and biased reasoning survived increasingly stringent demands of this kind (the conjunction fallacy is a case in point), sometimes not (pseudodiagnosticity illustrates). Either way, a significant amount of theoretical and/or experimental insight has been achieved. We conclude that normative concerns about diagnoses of (ir)rationality can retain a legitimate and constructive role for the psychology of reasoning.

## Footnotes

^1^We emphasize that here we are not committed in any way to the idea of T as a “computational model” or a “theory of competence,” as they are often understood. Such a move would risk to blur our current analysis (we concur with Evans and Elqayam, 2011: 277, and others on at least this much). Of course, T will be a formal system—say, classical probability theory. But, according to (i), in order for a diagnosis of irrationality to hold, T has to be taken as having normative force, namely, with an additional overarching claim that a rational agent ought to comply with its principles.
